# Investigating structure–property relationships of biomineralized calcium phosphate compounds as fluorescent quenching–recovery platform

**DOI:** 10.1098/rsos.170877

**Published:** 2018-02-07

**Authors:** Liuzheng Wang, Xiang He, Wei Zhang, Yong Liu, Craig E. Banks, Ying Zhang

**Affiliations:** 1 College of Science, Huazhong Agricultural University, Wuhan 430070, People's Republic of China; 2 Research Institute of Powder Metallurgy, Central South University, Changsha 410083, People's Republic of China; 3 Wuhan Institute of Marine electric Propulsion, Wuhan 430064, People's Republic of China; 4 Faculty of Science and Engineering, Manchester Metropolitan University, Chester Street, Manchester M1 5GD, UK

**Keywords:** biomineralization, calcium phosphate compounds, 8-hydroxy-2-deoxy-guanosine, fluorescent quenching–recovery

## Abstract

The structure–property relationship between biomineralized calcium phosphate compounds upon a fluorescent quenching–recovery platform and their distinct crystalline structure and surficial functional groups are investigated. A fluorescence-based sensing platform is shown to be viable for the sensing of 8-hydroxy-2-deoxy-guanosine in simulated systems.

## 1. Introduction

DNA damage plays a major role in mutagenesis, carcinogenesis and ageing [1–5] which arises due to exposure to exogenous chemicals or from metabolic, endogenous processes [6]. As the most reactive free radical, hydroxyl radical (radical ·OH) interacts with DNA (guanine) leading to the formation of 8-oxo-2′-deoxy-guanosine (8-oxo-dG) (also known as 8-OH-dG), which is widely used as a biomarker for oxidatively induced DNA damage [7–9]. For its determination, the 8-OH-dG levels are most commonly evaluated by high-performance liquid chromatography (HPLC), enzyme-linked immunosorbent assays (ELISA) or competitive enzyme immunoassay (EIA) kit [10–13]. However, these methods usually depend on complicated procedures and expensive equipment. The development of a detection platform with lower costs and portability has significant potential to increase its application base.

Functional micro-/nanomaterials have shown their unique application in tissue engineering, drug delivery and biosensing [14,15], and this is where biominerals demonstrate extraordinary structure−property relationships compared to their non-biogenic counterparts [16,17]. Inspired by nature, unique, effective biominerized strategies have been exploited to fabricate functional materials with intricate structures [18–20]. Among various biominerals, hydroxyapatite (HAp) is well known to be the main inorganic constituents of natural hard tissue such as bone and teeth, and has been extensively applied in bone implants and related fields [21,22]. The rapid development of material science and technology has meant that this material fabricated by artificial biomineralization has even proved increasingly promising in environmental toxic substance monitoring and disease-related biomarker detection [23–25], attributed to its controllable morphology and unique three-dimensional networks of crystalline structure. In our previous research, we have proposed a fluorescence-quenching platform based on the biomineralized HAp substrate from seashell (*Colossal False Fusus*) and applied this successfully to tumour cell identification [26].

**Scheme 1.**
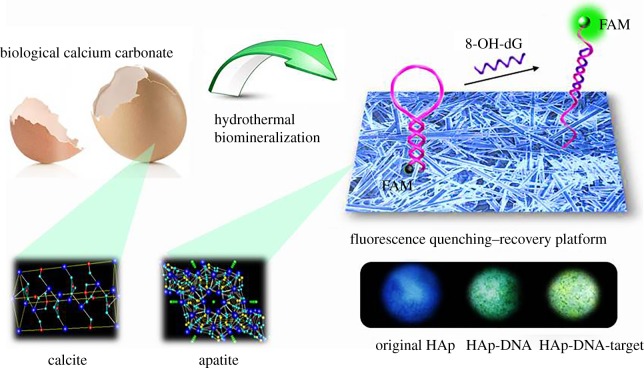
Schematic of the biomimetic platform for the detection of 8-OH-dG and the quenching–recovery conversion process.

In this paper, we report the synthesis of HAp via using four natural calcium carbonate biomasses, namely: seashell, conch, eggshell and coral, all of which are commonly encountered in our daily life. The structure−property relationships compared to their non-biogenic counterparts of original biomass are explored upon their florescence properties. A fluorescence quenching–recovery platform determination platform was constructed using a classical DNA beacon strategy and used for the sensing of 8-OH-dG in simulated system to achieve a convenient detection strategy via a simple colour differentiation (scheme 1). The insight into the structure–property relationship will enable HAp's broader application in clinical DNA damage determination.

## 2. Results and discussion

**Figure 1.**
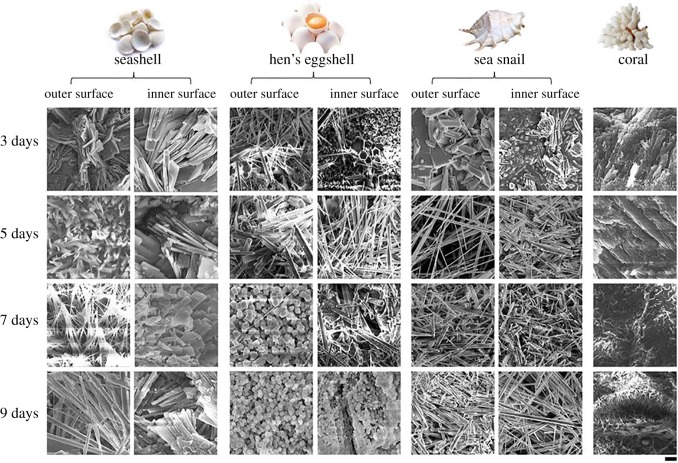
Morphology evolution of (*a*) seashell (*Nemocardium exasperatum*), (*b*) hen's eggshell, (*c*) sea snail (smooth spider conch) and (*d*) white coral after treated by hydrothermal biomineralization at 160°C for 3–9 days, respectively. The outer and inner surfaces were examined separately using SEM. Scale bar, 5 µm.

The shells of the biomass were treated as described in the experimental section (described in detail within the electronic supplementary material) to produce the fluorescence sensing platforms. In brief, the shells of the four natural calcium carbonate biomasses: seashell, conch, eggshell and coral were cut into approximately 1 × 1 cm pieces and ultrasonically cleaned. These pieces were then placed into autoclaves containing 80 ml of NH_4_H_2_PO_4_ solution (0.12 g ml^−1^) and heated at 160°C for 3 to 9 days. For visualization of these surface/substrates, the surface closest to the membrane was marked as ‘inner surface' while the outside was defined as ‘outer surface'. The morphology of all the samples was observed to be within the range of micrometres with both the outer and inner surfaces of the original shells experiencing a series of morphology evolutions during this hydrothermal treatment process, as clearly illustrated within figure 1. It can be noted that the evolution of this process and the resultant structural morphology is diverse in each sample. In the case of the seashell sample, the morphology of outer surface transformed from that of sheets (3 days) to threads (7 days) and then consequently wires (9 days), while the inner surface maintained sheets during this time period. On the other hand, in the case of hen's eggshells, both the outer and inner surfaces were generated from wires to that of particle-like morphology. For sea snail shells, the sheets observed in 3 days' samples developed into wires when the time extended to 5–9 days for both surfaces. Moreover, different from the first three samples, the morphology of coral maintained sheet-like formations during 3–7 days, but transformed to threads after a further 2 days.

In the case of calcium phosphate compounds, the Ca : P ratio roughly corresponds to the crystalline phase composite. EDAX characterization was performed in order to investigate the evolution of elemental composition (see electronic supplementary material, figure S1) and the corresponding Ca : P values were calculated and summarized within electronic supplementary material, table S1, which also reflects the distinguished transformation between the outer and inner surfaces. It can be inferred that these evolution morphological differences among these biomass shells were closely related to their distinct organic composition even for the same piece of material.

**Figure 2.**
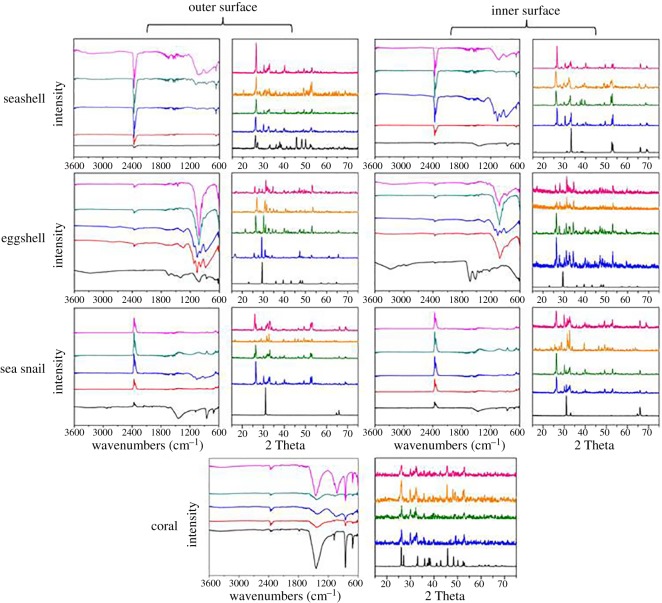
The functional groups on the surfaces (ATR-FTIR) and crystalline phase composition (XRD) of original biomass shells before (0 day) and after hydrothermal biomineralization for 3 ∼ 9 days. All the spectroscopies from bottom to top corresponding to 0, 3, 5, 7 and 9 day samples, respectively.

In order to characterize the superficial functional groups and crystalline phase composition of all the biomass shells, attenuated total reflectance Fourier transform infrared spectroscopy (ATR-FTIR) and X-ray diffraction (XRD) measurements were performed (figure 2). This analysis revealed that for both the surfaces of all the substrates before bonding with DNA, the characteristic bands at 1100–1000 cm^−1^ represent the phosphate group (*ν*3 triply asymmetric stretching mode of the P–O bond) and approximately 963 cm^−1^ (*ν*1 symmetric stretching mode of the P–O bond) for both outer and inner surface. Bands at approximately 3300 and 1631 cm^−1^ are due to O–H stretching and *ν*2 (H–O–H) bending modes of lattice water molecule. The band at 896–900 cm^−1^ can be attributed to the P-OH deformation indicating the protonation of the phosphate groups [26]. Additionally, bands in the 1630–1191 cm^−1^ region indicate the existence of carbonate groups, suggesting their incorporation into the crystal structure, which is possibly due to the absorption of carbon dioxide from the air during the treatment time.

XRD was also used in order to help characterize the material where the difference among these spectroscopy curves could be attributed to the evolution of crystalline composition during a holding/treatment time from 3 to 9 days. Detailed identification of crystalline structural parameters are listed in electronic supplementary material, table S2. It can be observed that the main transform process for all the substrates was calcite/aragonite (CaCO_3_) → brushite/monetite(CaHPO_4_) → hydroxyapatite (Ca_5_(PO_4_)_3_OH) → monetite/whitlockite (CaHPO_4_), which experiences a nucleation dissolution–recrystallization–self-assembly process, consistent with our previous report [27]:

Step 1 $$\begin{array}{rl} & \text{CaC}{\text{O}}_{3}+\text{ N}{\text{H}}_{4}{\text{H}}_{2}\text{P}{\text{O}}_{4}\to \text{CaHP}{\text{O}}_{4}+\text{N}{\text{H}}_{3}+\text{C}{\text{O}}_{2}+{\text{H}}_{2}\text{O,} \end{array}$$

Step 2 $$\begin{array}{r} 2\text{CaCO}3+3\text{CaHP}{\text{O}}_{4}\to \text{C}{\text{a}}_{5}{\left(\text{P}{\text{O}}_{4}\right)}_{3}\text{OH}+2\text{C}{\text{O}}_{2}+{\text{H}}_{2}\text{O} \end{array}$$

Step 3 $$\begin{array}{r} \;\text{and}\;\text{C}{\text{a}}_{5}{\left(\text{P}{\text{O}}_{4}\right)}_{3}\text{OH}+2\text{N}{\text{H}}_{4}{\text{H}}_{2}\text{P}{\text{O}}_{4}\to 5\text{CaHP}{\text{O}}_{4}+2\text{N}{\text{H}}_{3}+{\text{H}}_{2}\text{O}. \end{array}$$

**Figure 3:**
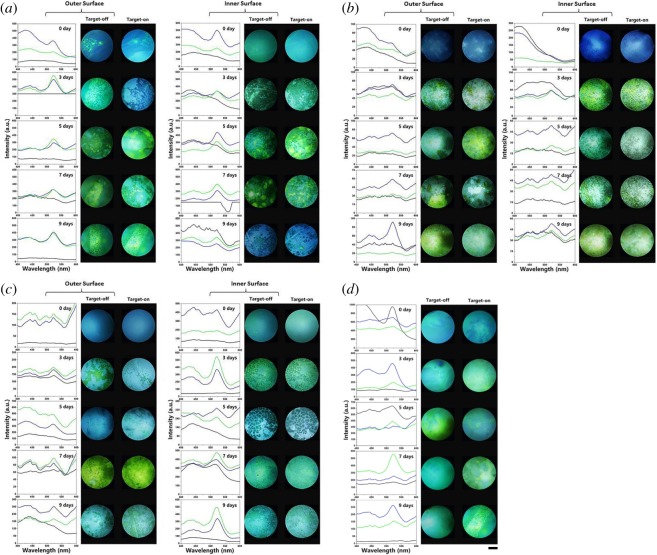
The photoluminescence (PL) spectroscopies and inversed fluorescence microscope (IFM) images for detection of 8-OH-dG by the proposed platforms constructed by (*a*) seashell, (*b*) hen's eggshell, (*c*) sea snail and (*d*) coral before (0 day) and after hydrothermally treated for 3 ∼ 9 days. Dark line, original surface; blue line, DNA modified surface without target; green line, DNA modified surface with target. Scale bar, 200 µm.

Next, the fluorescence properties of these as-prepared materials were characterized by grafting 5′FAM (carboxyfluorescein)-modified DNA beacon on the surface of the HAp materials/substrates with the DNA sequence specifically designed (by the supplying company) to be capable of hybridizing with 8-OH-dG, as summarized within (scheme 1). Herein, the photoluminescence (PL) spectra and IFM (immunofluorescence microscopy) imaging techniques were adopted simultaneously to verify the detected results, as shown in figure 3. Such work is comparable to the detection of 8-OH-dG using a nanopore sensing approach [28]. Similar to our previous report [28], it was expected to note that the as-prepared platform proposed here also shows distinct fluorescence quenching–recovery properties. One the one hand, no positive signal could be found either on the outer or the inner surface of untreated material whenever the targeted 8-OH-dG existed in the system, because DNA beacons could not graft onto the raw calcium carbonate material. On the other hand, for certain 3–9 days' treated substrates, the colour of fluorescence signals turned from blue to green suggesting the DNA beacons were successfully grafted onto the substrates (quenching) and the 5′-fluorophore came away from the surfaces when the target 8-OH-dG bonded (recovery). However, not all the surfaces were capable of such quenching–recovery transformations. Among the as-prepared platforms, the observable quenching–recovery capability was only obtained by seashell (both the outer and inner surface) and hen's eggshell (the outer surface) after 5 days' treatment, as well as the coral after 7 days' biomineralization. By contrast, we can barely find the mentioned transformation on all the sea snail platforms.

We infer that the unique fluorescence properties would have some relationship with the inherent quality of the natural biomass. The electronic transition between the fluorophores and quencher occurring during the excited state lifetime of the fluorophores gives rise to dynamic quenching; we infer that the crystalline structure plays an important role in this rather than the morphological structures (see SEMs, figure 1). The XRD identification results suggest that CaHPO_4_ (PDF no. 09-0080 and PDF no. 75-1520) have more considerable quenching–recovery capabilities than Ca_5_(PO_4_)_3_OH or other calcium phosphate compounds (e.g. the outer/inner surface of seashells and outer surface of eggshells after 5 days' treatment, see electronic supplementary material, table S2). The negative response of sea snails is attributed to their different surficial functional groups than the other three samples. From figure 2, the reversed peaks at approximately 2400 cm^−1^ in ATR-FTIR spectra of sea snails are speculated to be due to their fluorescence reflection property in this area. Another likely reason speculated to be associated with the surficial functional groups (as analysed and presented within figure 2), which was verified by the ATR-FTIR spectra of sea snail substrates with negative responding signals.

## 3. Conclusion

We have reported a hydrothermal biomineralization strategy for the fluorescent discrimination sensing of 8-OH-dG by quenching–recovery conversion. We have demonstrated that the appropriate crystalline structure and surficial functional groups are key factors for constructing a platform with fluorescent quenching–recovery capabilities by investigating the duration time and composition of biomass shells. It is revealed that the as-prepared substrates mainly consisting of CaHPO_4_ have more considerable fluorescence quenching–recovery capability than that constructed of Ca_5_(PO_4_)_3_OH or other calcium phosphate compounds. The outer/inner surface of seashells and outer surface of eggshells after 5 days' treatment exhibit distinct expected properties and can be used for fluorescent discrimination sensing of 8-OH-dG. This work forms the basis of extending further HAps application into clinical detection as a biomarker of real DNA damage samples.

## Supplementary Material

Supporting Information

## Data accessibility

Data used in this paper can be found in the electronic supplementary material.
