# Supplementary material for: Investigating structure–property relationships of biomineralized calcium phosphate compounds as fluorescent quenching–recovery platform
Source: R Soc Open Sci. 2018 Feb 7;5(2):170877. doi: 10.1098/rsos.170877 (PMC5830716; doi:10.1098/rsos.170877)
Supplement: Supporting Information [file rsos170877supp1.docx]

**Supporting Information for the following paper:**

**Investigating Structure-Property Relationships of bio-mineralised calcium phosphate compounds as fluorescent quenching-recovery platform**

Liuzheng Wang ^a^, Xiang He ^b^, Wei Zhang ^c^, Yong Liu ^b^, Craig E. Banks ^d,*^ and Ying Zhang ^a,*^

*^a.^ College of Science, Huazhong Agricultural University, Wuhan 430070, P.R.China.*

*^b.^ Research Institute of Powder Metallurgy, Central South University, Changsha 410083, P.R.China*

*^c.^ Wuhan Institute of Marine electric Propulsion, Wuhan 430064, P.R. China.*

*d. Faculty of Science and Engineering, Manchester Metropolitan University, Chester Street, Manchester M1 5GD, UK.*

**Experimental details**

*Fabrication of HAp by hydrothermal biomineralization strategy*

All reagents were purchased from Acros and used without any purification. All the solutions were prepared using ultra-pure water (18.2 MΩ) from Millipore Synergy system (AMETEK, UK). The shell of Smooth Spider Conch (*Lambis lambis*), *Nemocardium exasperatum* and hen’s egg, as well as white coral was broken into ~1 cm × 1cm pieces and ultrasonic cleaned with ultra-pure water and then anhydrous ethanol for 30 minutes in both cases. After being air-dried at 40 ^°^C for 2 hours, the shell pieces were added into autoclaves respectively containing 80 mL of NH_4_H_2_PO_4_ solution (0.12 g/mL). The autoclaves were then sealed and heated at 160 °C in a thermostat for 3 to 9 days. After this step, the products were filtered and alternately washed with ultra-pure water and anhydrous ethanol.

These samples were air-dried at 60 ^°^C for 12 hours, after which they were characterized with Field Emission Scanning Electron Microscope (FESEM; NOVA NANOSEM 230) equipped with EDAX Genesis, and X-Ray Diffraction meter (XRD), in order to identify the evolution of morphology, element composition and crystalline phrase for both the outer and inner surfaces after the duration from 3 to 9 days.

*Surface grafting with DNA molecule beacons*

The DNA molecule beacon used in this experiment was synthesized and modified by Sangon Biotech (Shanghai, China), with the sequence listed as below (5’-3’):

FAM-GCG GGC GAT CGG CGG GGG GTG CGT GCG CTC TGT GCC AGG GGG TGG GAC AGA TCA TAT GGG GGT GCT-NH_2_. FAM: carboxyflurescein.

In this work, all the solutions and vessels were used following a heat sterilization treatment. Firstly, the DNA molecule beacons were prepared into 100 μM stocking solution in 1×TE Buffer (pH 7.5~8.0; 10mM Tris, 1mM EDTA) and diluted to 1 μM for used by adding a certain volume of TE Buffer. Then the an as-prepared HAp substrate was immersed into 1 mL of the DNA molecule beacon solution and grafted in a Thermomixer comfort (Eppendorf) at 37 °C for 12 hours. Consequently, the DNA-modified HAp substrate was removed and washed with 1 × PBS (NaCl, KCl, Na_2_HPO_4_, KH_2_PO_4_) to remove the dissociative DNA.

*Construction of a fluorescence based sensing platform: application to 8-OH-dG detection*

The 8-hydroxy-2-deoxy-Guanosine (8-OH-dG) purchased from Sigma Aldrich (purity > 99%) was prepared into a 100 μg/mL stock solution in ultra-pure water and diluted to 1 μg/mL by adding a calculated volume of ultra-pure water. Then each as-prepared DNA-HAp substrate was immersed into 500 μL of target solution, and grafted in a Thermomixer comfort (Dragonlab) at 37 °C for 12 hours. Consequently, the target-DNA-HAp substrate was removed and washed with 1×PBS to remove the dissociative target.

For inspection of the substrates, they were taken out and loaded on a cleaned glass slide. Characterization was then performed via the use of an inversed fluorescence microscope (Olympus, IX71) by chromatic discrimination of the substrate surfaces before and after target capture. In order to confirm the determination results, the photoluminescence spectroscopies were measured by a spectrofluorometer (PerkinElmer, LS55) to provide supplementary evidence. The excitation source was a 450 W xenon lamp. An auto instrumental correction factor was applied to all PL spectra to correct for the wavenumber/wavelength dependent photomultiplier response and variation in output intensity from the lamp and grating efficiencies. Excitation and emission slit width were chosen about 10.0 nm for all the measurements, and the excitation wavelength was 325 nm.

1. **Element composition analysis**


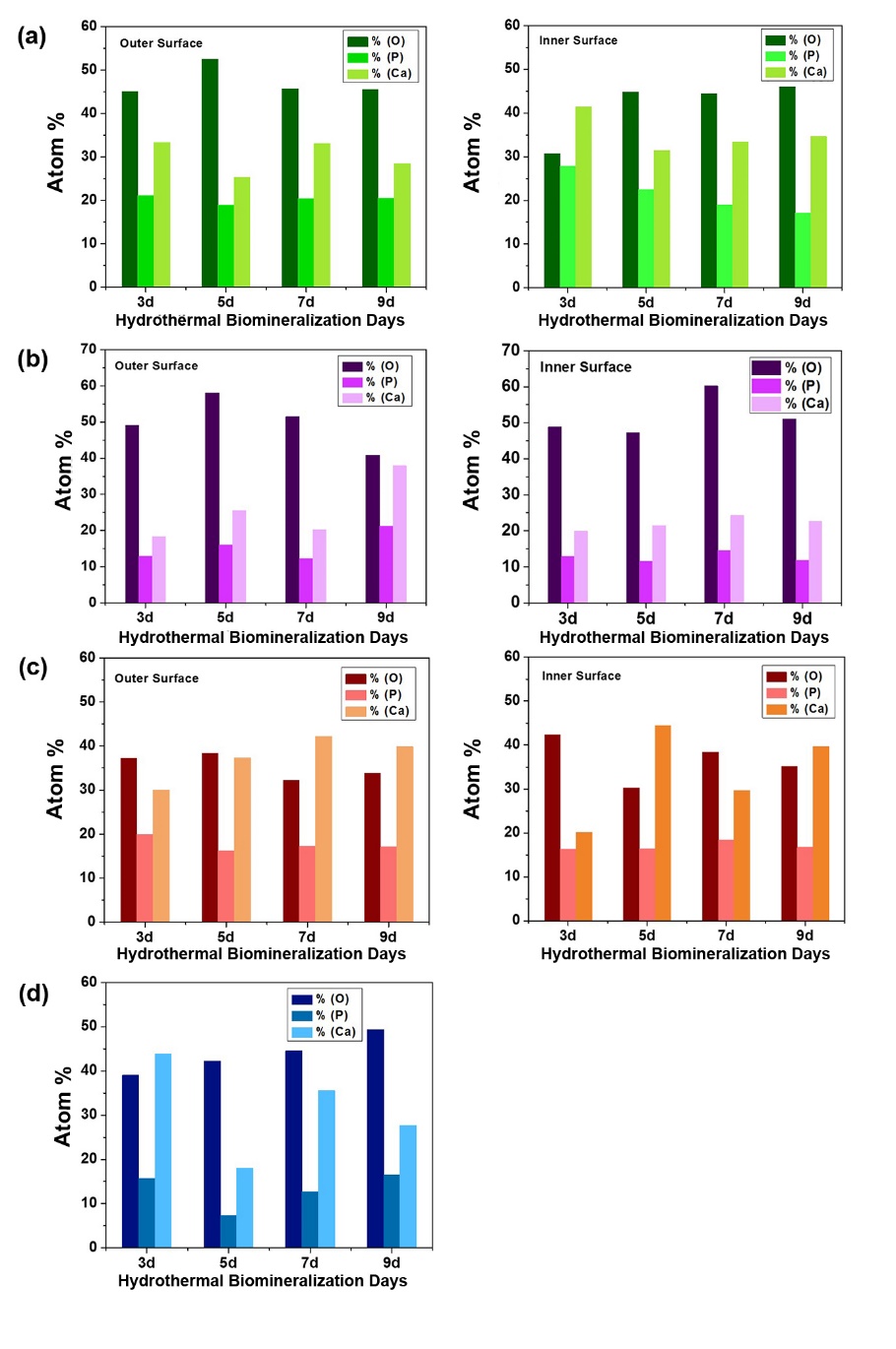


**Fig.S1** Evolution of element composition (Atom %) for (a) sea shell, (b) hen’s egg shell, (c) sea snail and (d) coral after hydrothermal biomineralization for 3 to 9 days.

**Table S1.** The calculated Ca:P ratio of biomass shells after hydrothermal biomineralization for 3 to 9 days

| Days | Sea shell | | Hen’s egg shell | | Sea snail | | Coral |
| --- | --- | --- | --- | --- | --- | --- | --- |
|  | *Outer* | *Inner* | *Outer* | *Inner* | *Outer* | *Inner* |  |
| 3 | 1.58 | 1.49 | 1.42 | 1.55 | 1.50 | 1.24 | 2.80 |
| 5 | 1.34 | 1.40 | 1.58 | 1.85 | 2.31 | 2.71 | 2.45 |
| 7 | 1.62 | 1.77 | 1.66 | 1.67 | 2.44 | 1.66 | 2.80 |
| 9 | 1.39 | 2.03 | 1.79 | 1.93 | 2.33 | 2.37 | 1.68 |

**Crystalline structural characterization**

**Table S2** The crystalline structural parameters of biomass shells after biomineralization for 3 to 9 days identified by XRD patterns. PDF: powder diffraction file (PDF) database.

| Biomass | Surface |  | Days | Main Phase | Chemical Formula | PDF# | a (Å) b (Å) | | c (Å) | α (º) | β (º) | γ (º) |  |
| --- | --- | --- | --- | --- | --- | --- | --- | --- | --- | --- | --- | --- | --- |
| Seashell | Outer |  | 3 | Brushite | Ca(PO_4_)_3_·2H_2_O | 09-0077 | 6.363 | 15.19 | 5.815 | 90.00 | 118.50 | 90.00 |  |
|  |  |  | 5 | Monetite | CaPO_3_(OH) | 09-0080 | 6.906 | 8.577 | 6.634 | 93.99 | 91.50 | 127.60 |  |
|  |  |  | 7 | Hydroxyapatite | Ca_10_(PO_4_)_6_(OH)_2_ | 54-0022 | 9.419 | 9.419 | 6.883 | 90.00 | 90.00 | 120.00 |  |
|  |  |  | 9 | Monetite | CaHPO_4_ | 70-1425 | 6.910 | 6.627 | 6.998 | 96.34 | 103.82 | 88.33 |  |
|  | Inner |  | 3 | Monetite | CaHPO_4_ | 70-1425 | 6.910 | 6.627 | 6.998 | 96.34 | 103.82 | 88.33 |  |
|  |  |  | 5 | Monetite | CaHPO_4_ | 75-1520 | 6.900 | 6.650 | 7.000 | 96.35 | 103.90 | 88.73 |  |
|  |  |  | 7 | Hydroxyapatite | Ca_8.8_(PO_4_)_6_(OH)_1.92_ | 86-1201 | 9.435 | 9.435 | 6.865 | 90.00 | 90.00 | 120.00 |  |
|  |  |  | 9 | Monetite | CaHPO_4_ | 70-1425 | 6.910 | 6.627 | 6.998 | 96.34 | 103.82 | 88.33 |  |
| Hen’s eggshell | Outer |  | 3 | Calcium Phosphate Hydrate | Ca_2_(P_4_O_12_) ·4H_2_O | 41-0483 | 7.667 | 12.889 | 7.144 | 90.00 | 107.00 | 90.00 |  |
|  |  |  | 5 | Monetite | CaPO_3_(OH) | 09-0080 | 6.906 | 8.577 | 6.634 | 93.99 | 91.50 | 127.60 |  |
|  |  |  | 7 | Hydroxyapatite | Ca_8.8_(PO_4_)_6_(OH)_1.92_ | 86-1201 | 9.435 | 9.435 | 6.865 | 90.00 | 90.00 | 120.00 |  |
|  |  |  | 9 | Whitlockite | Ca_2.933_H_0.014_(PO_4_)_2_ | 70-2065 | 10.439 | 10.439 | 37.375 | 90.00 | 90.00 | 120.00 |  |
|  | Inner |  | 3 | Hydroxyapatite | Ca_8.8_(PO_4_)_6_(OH)_1.92_ | 86-1201 | 9.435 | 9.435 | 6.865 | 90.00 | 90.00 | 120.00 |  |
|  |  |  | 5 | Monetite | CaHPO_4_ | 75-1520 | 6.900 | 6.650 | 7.000 | 96.35 | 103.90 | 88.73 |  |
|  |  |  | 7 | Hydroxyapatite | Ca_8.8_(PO_4_)_6_(OH)_1.92_ | 86-1201 | 9.435 | 9.435 | 6.865 | 90.00 | 90.00 | 120.00 |  |
|  |  |  | 9 | Whitlockite | Ca_2.933_H_0.014_(PO_4_)_2_ | 70-2065 | 10.439 | 10.439 | 37.375 | 90.00 | 90.00 | 120.00 |  |
| Sea snail | Outer |  | 3 | Monetite | CaHPO_4_ | 75-1520 | 6.900 | 6.650 | 7.000 | 96.35 | 103.90 | 88.73 |  |
|  |  |  | 5 | Monetite | CaHPO_4_ | 75-1520 | 6.900 | 6.650 | 7.000 | 96.35 | 103.90 | 88.73 |  |
|  |  |  | 7 | Hydroxyapatite | Ca_5_(PO_4_)_3_OH | 89-4405 | 9.426 | 18.856 | 6.887 | 90.00 | 90.00 | 119.97 |  |
|  |  |  | 9 | Hydroxyapatite | Ca_5_(PO_4_)_3_OH | 73-1731 | 9.400 | 9.400 | 6.930 | 90.00 | 90.00 | 120.00 |  |
|  | Inner |  | 3 | Monetite | CaHPO_4_ | 70-1425 | 6.910 | 6.627 | 6.998 | 96.34 | 103.82 | 88.33 |  |
|  |  |  | 5 | Monetite | CaHPO_4_ | 75-1520 | 6.900 | 6.650 | 7.000 | 96.35 | 103.90 | 88.73 |  |
|  |  |  | 7 | Calcium Hydrogen Phosphate Hydroxide | Ca_9_HPO_4_(PO_4_)_5_OH | 46-0905 | 9.441 | 9.441 | 6.881 | 90.00 | 90.00 | 120.00 |  |
|  |  |  | 9 | Monetite | CaHPO_4_ | 75-1520 | 6.900 | 6.650 | 7.000 | 96.35 | 103.90 | 88.73 |  |
| Coral |  |  | 3 | Calcium Cyclotetrahosphate Tetrahydrate | Ca_2_(P_4_O_12_) ·4H_2_O | 50-0582 | 10.107 | 10.450 | 13.651 | 90.00 | 109.42 | 90.00 |  |
|  |  |  | 5 | Calcium Cyclotetrahosphate Tetrahydrate | Ca_2_(P_4_O_12_) ·4H_2_O | 50-0582 | 10.107 | 10.450 | 13.651 | 90.00 | 109.42 | 90.00 |  |
|  |  |  | 7 | Monetite | CaHPO_4_ | 75-1520 | 6.900 | 6.650 | 7.000 | 96.35 | 103.90 | 88.73 |  |
|  |  |  | 9 | Calcium Cyclotetrahosphate Tetrahydrate | Ca_2_(P_4_O_12_) ·4H_2_O | 50-0582 | 10.107 | 10.450 | 13.651 | 90.00 | 109.42 | 90.00 |  |
